# Effect of leptin on the growth and expression of STAT3 in yak mammary epithelial cells

**DOI:** 10.14202/vetworld.2022.2141-2150

**Published:** 2022-09-07

**Authors:** Baoxia Dong, Sidra Mehran, Yuying Yang, Haixia Jing, Lin Liang, Xiaoyu Guo, Qinwen Zhang

**Affiliations:** 1 Department of Animal Medicine, College of Agriculture and Animal Husbandry, Qinghai University, Xining, China; 2 Department of Biotechnology, Kunlun College, Qinghai University, Xining, China

**Keywords:** JAK2, leptin, mammary epithelial cells, *STAT3*, yak

## Abstract

**Background and Aim:**

Leptin (LEP) is an autocrine and paracrine factor produced by the fat pad and acinar epithelial cells of the breast. This study aimed to investigate the effects of LEP on yak mammary epithelial cells (YMECs) and the expression of STAT3. In addition, we evaluated the possible effects of prolactin (PRL) on the function of LEP.

**Materials and Methods:**

The YMECs were treated with 0, 50, 100, 200, 400, and 800 ng/mL LEP for 48 h in the absence of PRL and the presence of 500 ng/mL PRL. The growth activity of YMECs was measured using the cell counting kit-8 assay. The changes in the lactation signaling pathway-related factor STAT3 were detected at the mRNA, protein, and protein phosphorylation levels using the reverse transcriptase-quantitative polymerase chain reaction and Western blotting. To explore whether LEP affects the activation of STAT3 through JAK2/JAK3 in YMECs, the JAK2/3 signaling pathway inhibitor AG490 was used at a fixed concentration of LEP.

**Results:**

Each concentration of LEP significantly promoted the expression of STAT3 mRNA (p < 0.05) in YMECs in the presence of PRL. In the absence of PRL, all concentrations of LEP were found to inhibit the expression of the STAT3 protein (p < 0.05). The expression of the STAT3 protein in YMECs was found to first increase followed by a decrease with an increase in the concentration of LEP. In addition, the phosphorylation level of STAT3 increased in all groups, except the 100 ng/mL concentration group. The STAT3 phosphorylation trend and protein expression were different, such that the level of protein phosphorylation was higher than that of the STAT3 protein (p < 0.05). The addition of AG490 reduced the expression of the STAT3 mRNA, STAT3 protein, and STAT3 phosphorylation in the LEP and LEP + PRL groups.

**Conclusion:**

Altogether, the results indicated that different concentrations of LEP exerted varying effects on the growth of YMECs and the expression of STAT3, and the activity of STAT3 was primarily activated by JAK2. The addition of LEP can effectively inhibit the downregulation of the JAK2/STAT3 signal pathway by AG490, mitigate its inhibitory effect on the proliferation of YMECs, and reduce apoptosis. We believe that these findings will provide a theoretical and experimental basis for future research in this field.

## Introduction

Yaks primarily inhabit the Qinghai-Tibet Plateau of China [1–3]. They are well-adapted to harsh environmental conditions such as low oxygen, low pressure, and extreme cold. Yaks are reared for economical products such as milk, meat, wool, and cashmere [4, 5]. In addition, yak milk is a natural product unique to the plateau, with high quality and high nutritional values. It is rich in functional and bioactive ingredients that are critical for antioxidation, regulation of the intestinal microflora, immune regulation, and anti-inflammation [1, 6–9]. Therefore, it highly caters to the expectations of consumers for high-quality dairy products.

However, the grazing environment, feeding methods, and various characteristics are known to relatively decreasing the yield of yak milk [10–12], thereby significantly limiting the economic status and product development in the market. Therefore, exploring the related factors affecting the growth and protein synthesis of yak mammary epithelial cells (YMECs) in detail is highly significant. Leptin (LEP) is primarily synthesized and secreted by adipocytes; however, the localization of its receptor mRNA transcripts by *in situ* hybridization indicated that LEP and its receptor transcripts are expressed explicitly in the mammary gland acinar epithelial cells. The LEP can be used as the autocrine and paracrine mediator of mammary gland metabolism. It maintains the activity of the mammary gland acinar epithelial cells during lactation [13], signifying its biological importance in the mammary gland [14]. The LEP primarily activates the JAK-STAT5/STAT3 signal transduction pathway during lactation [15]. This pathway is not only related to cell growth, proliferation, and differentiation but also participates in the apoptosis and degeneration of mammary epithelial cells (MECs) [16]. The MECs cultured *in vitro* demonstrated that MEC could secrete LEP and promote self-renewal upon LEP stimulation. A combination of prolactin (PRL) hormone and MEC improved the secretion of cow casein and promoted the proliferation of MECs [1, 17].

At present, there are many researches on LEP in the mammary gland, majorly focusing on tissue distribution [3, 18]. However, very little is known about the effect of exogenous LEP on MECs, signal transduction, and the underlying molecular mechanism, particularly with YMECs as a carrier. To assess the effect of LEP on the expression of STAT3, this study detected the changes in the growth activity and signal pathway-related factor STAT3 at the mRNA, protein, and phosphorylated protein levels in YMECs at different concentrations of LEP, including 0, 50, 100, 200, 400, and 800 ng/mL, using the cell counting kit-8 (CCK-8) method, reverse transcriptase-quantitative polymerase chain reaction (RT-qPCR), and Western blotting. Furthermore, AG490, a JAK2/3 signal pathway inhibitor, was used at a specific LEP concentration to explore whether LEP affects the activation of STAT3 in YMECs and the proliferation of YMECs through JAK2/JAK3. We believe that the findings of this study will provide certain theoretical support for the coordinated interaction of PRL, providing a basis for further studies in the later stage.

## Materials and Methods

### Ethical approval

All procedures were in compliance with the national laws and regulations for animal experimentation and performed in accordance with the guiding principles of the Qinghai University Animal Care Committee for the care and use of experimental animals (Approval no. IACUC, SL-2021020).

### Study period and location

The study was conducted from March 2021 to December 2021 in the State Key Laboratory of Sanjiangyuan Ecology and Plateau Agriculture and Animal Husbandry, Qinghai University, Xining City, Qinghai Province. The laboratory was jointly built by the Qinghai Provincial People’s government and the Qinghai provincial Ministry of Science and Technology. The cells used in this study were the primary YMECs preserved in our laboratory. All cells were identified by molecular phenotype and function.

### Effects of different concentrations of LEP on the growth of the YMECs

Before cell inoculation, blank holes were set as follows and the growth medium was added to each well [1 mg/mL Insulin-Transferrin-Selenium (Sigma, USA), 200 ng/mL hydrocortisone (Sigma), 10 ng/mL epidermal growth factor (Sigma) fetal bovine serum, penicillin, streptomycin, and Dulbecco’s Modified Eagle Medium/F12 culture medium]. The blank well contained the growth medium (acellular) and PRL (PeproTech) (–/+). The experimental Group I had the growth medium and LEP (Sigma), whereas well II was cultured with medium, LEP, and PRL. The LEP concentrations were set to 0, 50, 100, 200, 400, and 800 ng/mL with six multiple wells set for each experimental concentration.

The YMECs were collected and treated with 2 × 10^4^ cells/mL and inoculated in 96-well plates. Next, YMECs of the fourth-generation yak in the logarithmic growth stage were digested with 0.25% trypsin (Sigma). Ten microliters of CCK-8 (Boster, Wuhan, China) were added to the 96-well plates daily; after incubation for 2.5 h, the optical density (OD) value of each well was measured at 450 nm using a microplate reader (Thermo Varioskan Lux Reader). The cells were counted continuously until they covered the bottom of the hole. Next, the growth curve of YMECs was drawn with the culture time (d) on the X-axis and OD value on the Y-axis.

The relative OD values of cells under different concentrations of LEP were counted at 24, 96, and 128 h of culture. Cells with an LEP concentration of 0 ng/mL were used as the control; these reflected the relative growth of cells in the initial stage, logarithmic growth stage, and stable stage of inoculation under different concentrations of LEP.

### Cell processing

We selected and digested fourth-generation YMECs in the logarithmic growth stage using 0.25% trypsin. Next, the unified concentrated cells were collected, centrifuged, and inoculated in the same amount in the cell culture bottle and marked as PRL (–) + LEP 0, 50, 100, 200, 400, and 800 ng/mL groups; PRL (+) + LEP 0, 50, 100, 200, 400, and 800 ng/mL groups, that is, each group was divided into 12 treatment groups with three replicates within each treatment group. Cells with an LEP concentration of 0 ng/mL were used as controls. The culture medium was discarded after a cell density of around 80% was attained. The cells were hydrated with phosphate-buffered saline 2–3 times, and the LEP culture medium of the corresponding treatment group was added to each culture bottle. The follow-up experiment operation was performed after culturing for 48 h.

### Real-time fluorescent quantitative polymerase chain reaction (RT-qPCR) analysis of gene expression

The PCR was used to analyze the gene expression through quantitative characterization of cell samples. To extract the total RNA, three bottles of purified fifth-generation YMECs were selected according to the instructions provided in the manual (RNA Extraction Kit, Tiangen, Beijing, China). Next, these samples were used to detect the purity, concentration, and RNA integrity using the Ultramicro ultraviolet spectrophotometer (NanoPhotometer NP80, Implen, Germany) and 1% agarose gel electrophoresis. RNA (100 ng/mL) was reverse transcribed into cDNA using the reverse transcription reagent (Tiangen) at 42°C for 15 min and 95°C for 3 min. The reaction products were stored at −20°C until further use.

The primers were designed using the primer (Premier 5.0, Canada) according to the complete sequences of *STAT3* (GenBank accession number: XM_005899206.1) and *β-actin* (GenBank accession number: XM_005887322.2) genes recorded on GenBank. The sequence homology was analyzed using the blast function in the National Center for Biotechnology Information database to ensure primer specificity. The primers were synthesized by Beijing Liuhe Huada Gene Technology Co., Ltd., and the sequences are presented in Table-1.

**Table-1:** Primer sequences for RT-qPCR.

Gene	Primer sequence (5′–3′)	Temperature	Length (bp)
*β-actin*	Forward: TGATGATATTGCTGCGCTCG	61°C	153
	Reverse: TACGAGTCCTTCTGGCCCAT		
*STAT3*	Forward: TCTGGGCACAAACACGA	61°C	143
	Reverse: CGGTCACAATCAGGGAG		

RT-qPCR=Reverse transcriptase-quantitative polymerase chain reaction

Genetic characterization of cell samples was ascertained using a Roche LightCycler 480 II real-time fluorescent quantitative PCR instrument (Roche, Switzerland). According to the instructions provided in the fluorescent quantitative kit (Tiangen), the reaction mixture comprised 2 μL of cDNA, 0.6 μL of the forward and reverse primers, 10 μL of SuperReal PreMix Plus (2×), and 6.8 μL of ddH_2_O. The reaction conditions were 94°C for 5 s; 40 cycles of 95°C for 5 s, 61°C for 30 s, 72°C for 30 s; 95°C for 5 s, and 70°C for 60 s. The relative mRNA expression of genes was calculated using the 2^−ΔΔCT^ method and normalized to the value of *β-actin*.

### Detection of STAT3 protein and its phosphorylation expression by Western blotting

This study selected three bottles of YMECs for each treatment group. The whole protein was extracted according to the instructions described in the whole protein extraction kit manual (Keygen Biotech, Jiangsu, China). Afterward, the protein concentration was determined according to the instructions given in the bicinchoninic acid assay protein concentration determination kit (Beyotime, Shanghai, China). To calculate the total protein, the protein lysate was diluted to a uniform concentration, using the following formula: 4 × protein loading buffer (3:1). The denaturation condition was a 40 mL system, at 100°C for 5 min.

The separation gel and concentrated gel were prepared according to the molecular weights of b-actin, STAT3, and p-STAT3, and their protein expression was detected by Western blotting. After exposure to the enhanced chemiluminescence reagent, images were captured and recorded using a fluorescence imaging system (ProteinSimple FluorChem E, ProteinSimple, USA). The following primary antibodies were used: Anti-STAT3 antibody (1:1000), anti-p-STAT3 antibody (1:2000), and anti-beta-actin antibody (1:10000). The secondary antibody was used at a concentration of 1:5000.

### Effects of AG490 on the growth and STAT3 expression of YMECs

The concentration of the inhibitor was set to 25 ng/mL according to those reported in the previous studies [17, 19]. Based on the presence or absence of PRL and the expression of STAT3 in the above test, the LEP concentration was set to 200 ng/mL. Two types of culture media containing inhibitors (the major steps of other experiments were the same as before) were prepared and the action time was 48 h.

1. Prolactin free medium: LEP + subculture medium + inhibitor
2. Prolactin containing the medium: PRL (500 ng/mL) + LEP + passage medium + inhibitor.

Before cell inoculation, the blank well (containing hormone and growth medium only), control well (containing hormone, growth medium, and cells), and experimental well (containing hormone, growth medium, cells, and AG490) were prepared and the cell inhibition rate, mRNA, protein, and their phosphorylation levels were detected. The cell inhibition rate was calculated using the formula: (A_control_–A_experimental_)/(A _control_–A_blank_) × 100%. The other related operation steps were undertaken as previously described.

### Statistical analysis

Mean values generated from all individual data were statistically analyzed by one-factor variance analysis and multiple comparisons using Duncan’s Statistical Package for the Social Sciences 22.0 (IBM Corp., NY, USA). GraphPad Prism 9 (GraphPad) and origin 8.0 (Originlab, USA) were used to plot analysis results.

## Results

### Effects of different concentrations of LEP on the growth of YMECs

#### Effects of different concentrations of LEP on the characteristics of cell growth in the absence of PRL

**Figure-1:**
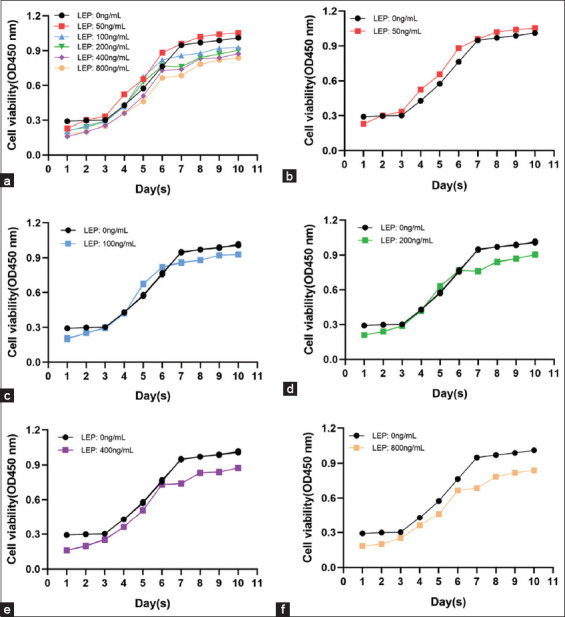
Growth curve of the yak mammary epithelial cells under different concentrations of leptin without prolactin. (a) Growth curve of the yak mammary epithelial cells at concentrations of 0, 50, 100, 200, 400, and 800 ng/mL leptin, (b) growth curve of the yak mammary epithelial cells at 0 and 50 ng/mL leptin concentration, (c) growth curve of yak mammary epithelial cells at 0 and 100 ng/mL leptin concentration, (d) growth curve of yak mammary epithelial cells at 0 and 200 ng/mL leptin concentrations, (e) growth curve of yak mammary epithelial cells at 0 and 400 ng/mL leptin concentration, and (f) growth curve of yak mammary epithelial cells at 0 and 800 ng/mL leptin concentration.

The YMECs were inoculated with different concentrations of LEP and cultured under the same conditions without PRL. Cells incubated with 0, 50, 100, 200, 400, and 800 ng/mL LEP maintained their growth characteristics. The growth curve demonstrated a characteristic “S” shape; cells entered the logarithmic growth phase on the 3^rd^ day (Figure-1).

**Figure-2:**
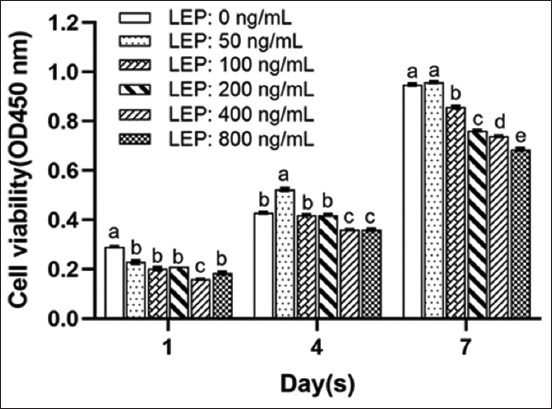
Cell activity of different concentrations of LEP at different times of culture without PRL. Single-factor analysis of variance within the group was used to detect the cell activity simultaneously (n = 4). Values are represented as means ± standard deviation (n = 4); means with different superscripts differ significantly (p < 0.05). LEP=Leptin, PRL=Prolactin.

The number of cells in the control group was found to be significantly higher than that in the experimental group (p < 0.05) (Figures-1a and 2). The cells demonstrated different proliferation efficiencies to varying concentrations with an increase in the culture time. On the 2^nd^ day, the number of cells at 50 ng/mL LEP increased rapidly and remained dominant later (Figures-1 and 2). After 4 days of culture, cells were treated with 100 ng/mL and 200 ng/mL LEP to maintain the same growth trend and entered the rapid proliferation phase. The number of cells was significantly higher than cells treated with 400 and 800 ng/mL LEP (p < 0.05). The growth was significantly inhibited on treatment with 400 and 800 ng/mL LEP (Figures-1 and 2). In the absence of PRL, the cell growth was inhibited within the 1^st^ day using different concentrations of LEP. With prolonged culture time, 50 ng/mL LEP promoted cell proliferation, and 100 ng/mL and 200 ng/mL LEP improved cell proliferation from the 4^th^ to 6^th^ day. However, cell proliferation was significantly inhibited with increased LEP concentration and culture time.

### Effects of different concentrations of LEP on growth characteristics of PRL cells

**Figure-3:**
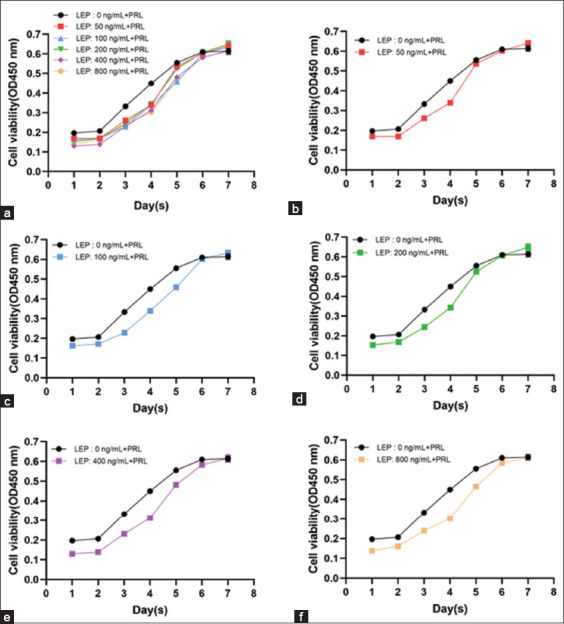
Growth curve of YMECs under different concentrations of LEP with PRL. (a) Growth curve of YMECs at LEP concentrations of 0, 50, 100, 200, 400, and 800 ng/mL after adding PRL, (b) growth curve of YMECs at LEP concentrations of 0 and 50 ng/mL after adding PRL, (c) growth curve of YMECs at LEP concentrations of 0 and 100 ng/mL after adding PRL, (d) growth curve of YMECs at LEP concentrations of 0 and 200 ng/mL after adding PRL, (e) growth curve of YMECs at LEP concentration of 0 and 400 ng/mL after adding PRL, and (f) growth curve of YMECs at LEP concentration of 0 and 800 ng/mL after adding PRL. LEP=Leptin, PRL=Prolactin, YMECs=Yak mammary epithelial cells.

**Figure-4:**
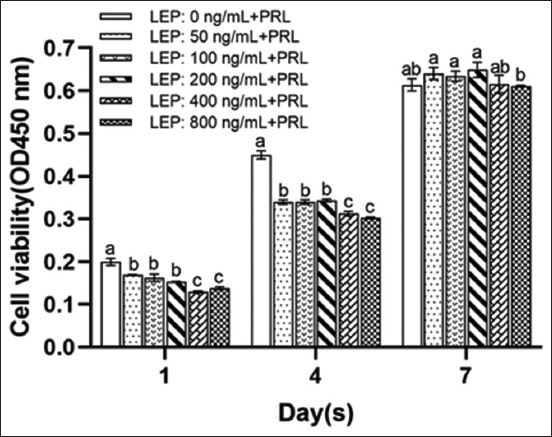
Cell activity at different concentrations of LEP at different culture times with PRL. The activity of cells cultured for 1, 4, and 7 days at LEP concentrations of 0, 5, 100, 200, 400, and 800 ng/mL. Means with different superscripts differ significantly (p < 0.05). LEP=Leptin, PRL=Prolactin.

In the presence of PRL, cells exposed to all LEP concentrations showed normal growth characteristics and an incubation period at an early stage of culture. The overall growth trend of cells cultured at five concentrations was similar, and the cells entered logarithmic growth phase on the 2^nd^ day (Figures-3a and 4).

The number of cells in 50, 100, 200, 400, and 800 ng/mL LEP groups on the first 4 days of culture was significantly lower than that in the control group (p < 0.05). The number of cells in each concentration group on the 6^th^ day of culture was similar; however, there was no significant difference in the number of cells on the 7^th^ day (p > 0.05) (Figures-3b-f and 4). This could be attributed to the rapid proliferation of cells in each concentration of LEP from the 4^th^ to 5^th^ day. Altogether, LEP inhibited the growth of YMECs in the presence of PRL; and the inhibition heightened with the increased concentration of LEP. However, cell proliferation rate could be effectively improved from the 4^th^ to 5^th^ day.

### Effects of different concentrations of LEP on STAT3 gene expression

#### Quality and purity detection of the total RNA

The purity of the extracted RNA was detected to be high, and the A_260_/A_280_ was found to be between 1.9 and 2.1. The electrophoresis results showed three visible bands, where the brightness of 28S was about twice that of 18S, indicating that the extracted RNA met the requirements of subsequent experiments.

#### Primer specificity

The primers for the *STAT3* gene and internal reference gene *β-actin* showed a single band with strong specificity, which met the experimental requirements and could be used in subsequent experiments.

#### Analysis of STAT3 mRNA expression under different concentrations of LEP

**Figure-5:**
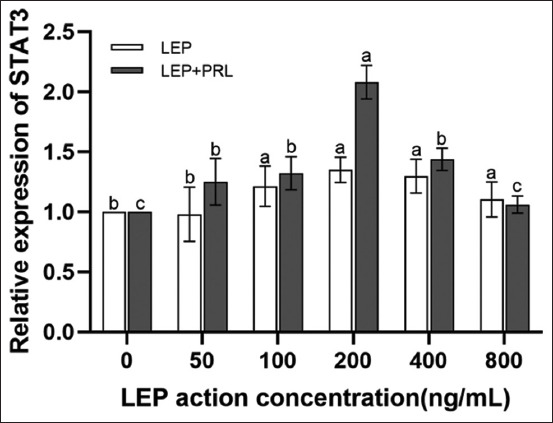
Expression characteristics of *STAT3* mRNA under different concentrations of LEP. Results of one-way analysis of variance for the effects of different concentrations of leptin on the mRNA expression of STAT3 (n = 3). Values are represented as means ± standard deviation (n = 3); means with different superscripts differ significantly (p < 0.05). LEP=Leptin.

The RT-qPCR results showed a positive correlation between LEP concentration and *STAT3* mRNA level in the absence of PRL (Figure-5). In the presence of PRL, different LEP concentrations promoted the expression of *STAT3* mRNA in YMECs, with the strongest effect at a concentration of 200 ng/mL (p < 0.05).

### Effects of different concentrations of LEP on the expression of STAT3 protein and its phosphorylation level

#### Effects of different concentrations of LEP on STAT3 protein and its phosphorylation

**Figure-6:**
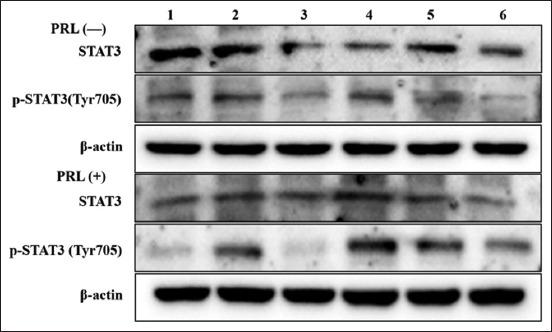
Enhanced chemiluminescence exposure results show STAT3 and its phosphorylated protein under different concentrations of LEP in PRL (–/+). Bands 1, 2, 3, 4, 5, and 6 represent LEP concentrations at 0, 50, 100, 200, 400, and 800 ng/mL, respectively. LEP=Leptin, PRL=Prolactin.

**Figure-7:**
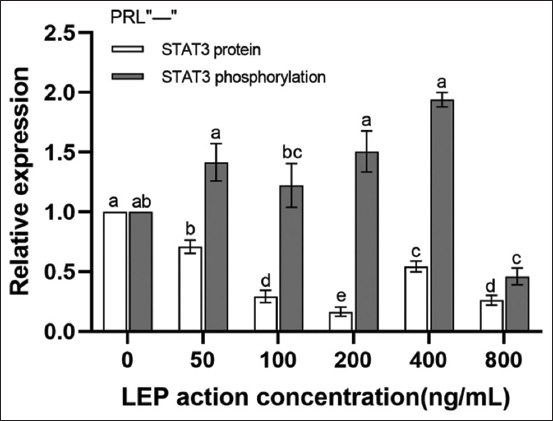
Expression of STAT3 protein and its phosphorylation level under different concentrations of LEP without PRL. The total STAT3 protein and its phosphorylation without PRL were detected by western blotting and analyzed quantitatively. Values are represented as means ± standard deviation (n = 3); means with different superscripts differ significantly (p < 0.05). LEP=Leptin, PRL=Prolactin.

**Figure-8:**
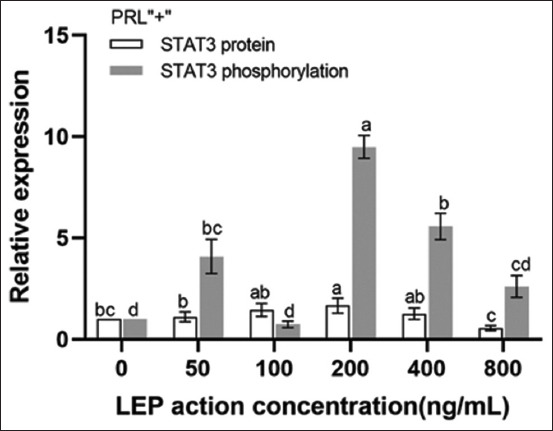
Expression of STAT3 protein and its phosphorylation in the presence of PRL at different concentrations of LEP. The total STAT3 protein and its phosphorylation after the addition of PRL were detected by Western blotting and analyzed quantitatively. The relative expression is the average value (n = 3), means with different superscripts differ significantly (p < 0.05). LEP=Leptin, PRL=Prolactin.

Western blotting (Figure-6) showed that in the absence of PRL, the expression of STAT3 protein was inhibited by all concentrations of LEP (p < 0.05). The most significant inhibition was evident at a concentration of 200 ng/mL (p < 0.05). The phosphorylation level of STAT3 was higher than that of STAT3, particularly at 200 ng/mL, where the level of STAT3 phosphorylation peaked and the level of total protein was the lowest (p < 0.05) (Figure-7). In the presence of PRL, the expression of STAT3 protein was found to first increase, followed by a decrease with an increase in LEP concentration. The expression of STAT3 protein in the 200 ng/mL concentration group was significantly higher than that in the 50 ng/mL concentration group (p < 0.05). The STAT3 protein in the 200 ng/mL concentration group was slightly higher than that in the 100 ng/mL and 400 ng/mL concentration groups (p > 0.05), which was significantly inhibited at a concentration of 800 ng/mL LEP (p < 0.05). The level of STAT3 phosphorylation was increased in all groups, except the 100 ng/mL concentration group. The STAT3 phosphorylation level was the highest when the concentration of LEP was 200 ng/mL (p < 0.05) (Figure-8).

#### AG490 inhibited the growth of YMECs

**Figure-9:**
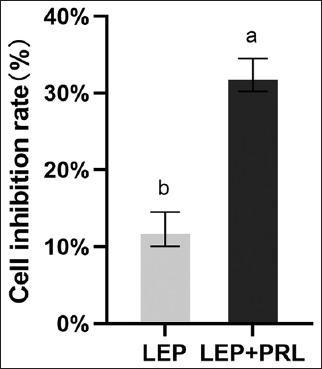
Growth inhibition effect of AG490 on the two groups of cells. The inhibition rate of AG490 was compared for cells with or without prolactin by an independent sample t-test as evident below. Means with different superscripts were found to differ significantly (p < 0.05).

The cells were cultured in PRL (–) and PRL (+) media containing 200 ng/mL LEP and 25 ng/mL JAK2/JAK3 signaling pathway inhibitor (AG490) for 48 h. Subsequently, the absorbance value of each group was measured at 450 nm using the CCK-8 assay, and the growth inhibition rates of the two groups of cells were calculated. The results showed significantly lower inhibition of cell growth in the PRL-containing group (p < 0.05) than that in the absence of PRL in the culture medium (Figure-9). This suggested that AG490 is a better inhibitor of the downstream factors and plays a regulatory role in cell growth in the presence of PRL. In addition, it indicated that LEP might not regulate the growth of YMECs through the JAK2/JAK3 signaling pathway.

#### Effect of AG490 on the expression of STAT3 mRNA in YMECs

**Figure-10:**
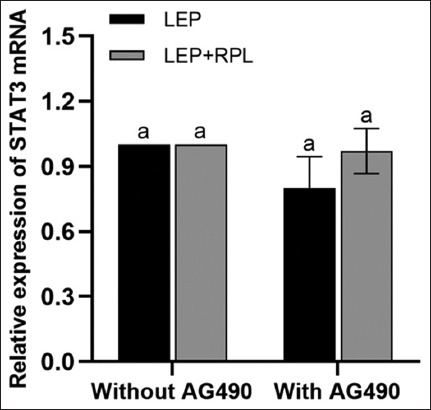
Effect of inhibitor on the expression of STAT3 mRNA. The effect of AG490 on the STAT3 mRNA expression in yak mammary epithelial cells was detected using real-time fluorescence quantitative PCR. The relative expression is the average value (n = 3), means with different superscripts differ significantly (p < 0.05). PCR=Polymerase chain reaction.

The RT-qPCR analysis revealed that the *STAT3* mRNA levels in the LEP group and LEP + PRL group decreased on adding AG490. However, no significant difference was observed compared to groups without AG490 (p > 0.05) (Figure-10), indicating that AG490 does not significantly affect the STAT3 transcription with or without PRL.

#### Effect of AG490 on STAT3 protein expression and phosphorylation in the YMECs

**Figure-11:**
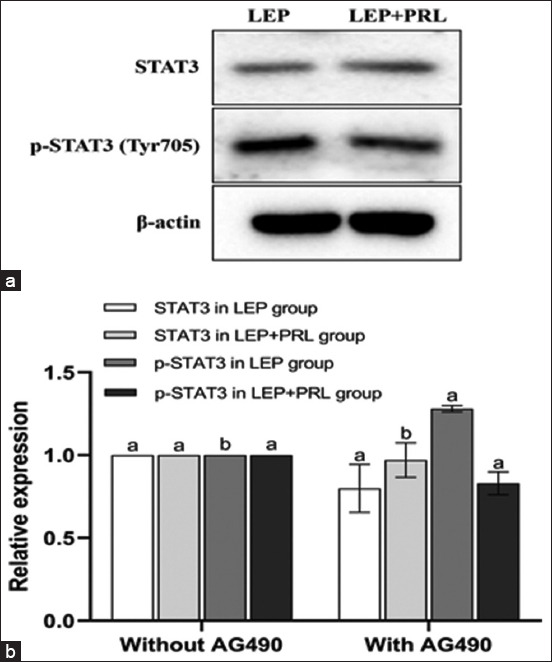
Effect of the inhibitor on the expression of STAT3 protein and its phosphorylation level. (a) The expression of STAT3 total protein and STAT3 phosphorylation was detected by Western blotting. (b) The expression of STAT3 total protein and STAT3 phosphorylation was analyzed quantitatively. The relative expression is the average value (n = 3), means with different superscripts differ significantly (p < 0.05).

Western blotting results showed that the expression of STAT3 protein in the LEP group and PRL + LEP group, and the phosphorylation of STAT3 in the PRL + LEP group reduced after culturing with AG490 for 48 h. Notably, the expression of the STAT3 protein decreased significantly in the presence of PRL (p < 0.05) (Figure-11), indicating that STAT3 activation can be mediated by JAK2 or JAK3 in the presence of PRL. Because JAK3 majorly exists in the myeloid or lymphoid cells, it also indicated that JAK2 is primarily activated in YMECs under the action of PRL. In the LEP group, the STAT3 phosphorylation level remained uninhibited and increased, illustrating that JAK2 is not the upstream factor required to activate STAT3 phosphorylation. It is speculated that following the inhibition of the JAK2/JAK3 activity, LEP could regulate the downstream phosphorylation of STAT3 through a different pathway.

## Discussion

The LEP is a growth factor that can stimulate the proliferation of different cell types [20]. It is primarily but not exclusively synthesized and secreted by the adipose tissue. The LEP is positively expressed in the breast myoepithelial cells and secretory epithelial cells. The secretory epithelial cells can synthesize LEP and transfer it from the blood, demonstrating that LEP directly regulates the development and function of the mammary gland [1].

Higher concentrations of LEP have been reported to induce cell proliferation *in vitro* than the normal circulating LEP levels [21]. At a low concentration, LEP affects cell proliferation in a time- and dose-dependent manner [22], interfering with the polypeptide growth factor signal and steroid receptor. The results showed the important role of PRL in the function of LEP in MECs growth. In the absence of PRL, a concentration of 50 ng/mL of LEP significantly affected cell proliferation. In contrast, the cell growth at different concentrations of LEP showed a similar inhibition in the presence of PRL. However, all of them entered the logarithmic growth phase in advance. On the 4^th^ day, the number of proliferating cells at 50 ng/mL and 200 ng/mL LEP increased slightly, followed by a rapid proliferation rate of each concentration group at a fixed concentration of PRL. This finding showed that LEP exerts a time-dependent effect on the proliferation of YMECs, which could be due to the synergistic effect of LEP on the expression of milk protein in the presence of PRL [23]. Silva *et al*. [24] reported that injecting a high concentration of LEP into the mammary gland of prepubertal heifers reduced the proliferation of MECs. Faggioni *et al*. [22] cultured the cow mammary gland tissue *in vitro* and treated it with different concentrations of LEP (0, 10, and 100 ng/mL) and PRL (0 and 1 mg/mL). In the absence of PRL, LEP did not promote the expression of the gene encoding the milk protein. In the presence of PRL, varying concentrations of LEP exerted different promoting effects, among which the strongest promoting effect was evident at a concentration of 100 ng/mL. Baratta *et al*. [25] confirmed LEP to promote the expression of milk protein in mammary epithelial HC11 cell lines with and without PRL; these results were consistent with the results of this study.

After activating the LEP receptor, the STAT3 molecules are recruited through its SH2 domain, and after tyrosine phosphorylation, these are transported to the nucleus as homodimers to induce specific gene expression. In general, STAT3 is essential for the apoptosis and degradation of MECs [26]; however, LEP might also promote cell proliferation by activating STAT3, thereby inducing cell differentiation [27] and mediating the expression of anti-apoptotic genes [28]. In this study, the *STAT3* mRNA increased at first and subsequently decreased at different concentrations of LEP in the presence of PRL, with the highest expression at 200 ng/mL. The STAT3 phosphorylation increased at 50 ng/mL and subsequently decreased after reaching the highest level at 200 ng/mL. This was consistent with the growth characteristics of MECs under different concentrations of LEP. Zhang *et al*. [29] reported that the levels of p-STAT3 increased significantly when mouse neuronal cells were treated with LEP (200 ng/mL). In addition, Pai *et al*. [30] observed that LEP significantly increased the phosphorylation of STAT3 in HaCaT gastric cancer, thereby promoting the proliferation of gastric cancer cells. This is in contrast to our study involving treatment of 100 ng/mL LEP to understand its effect on the levels of total STAT3 protein and its phosphorylation level, considering the concentration design, species differences, and cell characteristics. Moreover, LEP regulates mitochondrial oxidative stress and reduces apoptosis by affecting the phosphorylation of STAT3 [31]. Furthermore, our results indicate that p-STAT3 is related to the proliferation of anti-apoptotic YMECs. However, the specific mechanism between STAT3 phosphorylation and cell growth characteristics in YMECs remains unclear.

The JAK2 is preferentially activated in the LEP receptor signal transduction. In addition, the difference in the expression of protein and protein phosphorylation levels indicates the function of certain internal kinases. AG490, an inhibitor of the JAK2/JAK3 signaling pathway, was used to treat cells at 200 ng/mL LEP. The results indicated that in the presence of PRL, the phosphorylation level of STAT3 was decreased and the cell inhibition rate was higher. In the absence of PRL, the phosphate level increased and the cell inhibition rate decreased significantly. The expression of STAT3 protein was inhibited with or without PRL. This indicates that the phosphorylation of STAT3 is related to cell activity. The addition of LEP can effectively inhibit the downregulation of the JAK2/STAT3 signaling pathway by AG490, mitigate its inhibitory effect on the proliferation of YMECs, and reduce cell apoptosis. This finding also suggested that STAT3 could be partly induced by JAK2 activation with PRL, thus enhancing the induction and activation of JAK2 in the LEP signaling pathway [32, 33].

## Conclusion

In general, this study found that the addition of LEP to YMECs *in vitro* could affect the proliferation of YMECs and exerted a certain effect on *STAT3* mRNA, protein, and phosphorylation in YMECs. The addition of 50 ng/mL LEP significantly promoted the proliferation of YMECs. The protein phosphorylation level of STAT3 is related to cell activity. Furthermore, 50 ng/mL and 200 ng/mL LEP may promote the proliferation of YMECs by increasing the phosphorylation level of STAT3. After adding AG490, LEP reduced the apoptosis induced by AG490 by inhibiting the inhibition of JAK2/STAT3 by AG490.

In this study, the effects of different concentrations of LEP on STAT3 expression were discussed, and AG490 was used under a fixed concentration of LEP to explore whether LEP mainly affects STAT3 activation in YMECs through JAK2, thus enriching enrich the effects of LEP and LEP on the growth of plateau YMECs and provide theoretical support for the coordinated interaction between LEP and PRL. In addition, it provides a certain reference for future research. However, the specific mechanism between STAT3 phosphorylation and its effect on the growth characteristics of YMECs needs to be further studied.

## Authors’ Contributions

BD: She is a master’s student who did most part of the research, designed the study and experiments, collected data, performed the statistical analysis, and wrote the manuscript. SM: Designed the research, method, and collected data. YY and XG: Data collection and analysis. HJ and LL: Directed in method, data collection, and manuscript revision. QZ: Directed in method and data collection. All authors have read and approved the final manuscript.
